# Temperature-Dependent Raman Spectroscopic Study of the Double Molybdate KBi(MoO_4_)_2_

**DOI:** 10.3390/ma13235453

**Published:** 2020-11-30

**Authors:** Min Wang, Changhao Wang, Jian Wang, Liming Lu, Xiaoye Gong, Xiaohui Tang, Fu Zhang, Jinglin You

**Affiliations:** 1 State Key Laboratory of Advanced Special Steel, School of Materials Science and Engineering, Shanghai University, Shanghai 200444, China; wang_min@shu.edu.cn (M.W.); wang_jian@shu.edu.cn (J.W.); crusenicd@shu.edu.cn (X.G.); xhtang_gray@shu.edu.cn (X.T.); zhangfu@shu.edu.cn (F.Z.); 2 Key Laboratory of Infrared Imaging Materials and Detectors, Shanghai Institute of Technical Physics, Chinese Academy of Sciences, Shanghai 200083, China; ch14820189@shu.edu.cn; 3 CSIRO Mineral Resources, Technology Court, Pullenvale, QLD 4069, Australia; Liming.Lu@csiro.au

**Keywords:** high-temperature Raman spectroscopy, quantum chemistry ab initio calculation, structure of melt

## Abstract

In situ high-temperature Raman spectra of polycrystalline KBi(MoO_4_)_2_ were recorded from room temperature to 1073 K. Thermal stability of the monoclinic KBi(MoO_4_)_2_ was examined by temperature-dependent XRD. The monoclinic phase transformed into the scheelite tetragonal structure at 833 K, and then to the monoclinic phase at 773 K. Quantum chemistry ab initio calculation was performed to simulate the Raman spectra of the structure of KBi(MoO_4_)_2_ high-temperature melt. The experimental Raman band at 1023 K was deconvoluted into seven Gaussian peaks, and the calculated results were in good agreement with the experimental data. Therefore, the vibrational modes of Raman peaks of molten KBi(MoO_4_)_2_ were assigned. It was confirmed that the isolated structure of [Bi(MoO_4_)_2_]^−^ monomer, consisting of Mo^6+^ centers and Bi^3+^ sub-centers connected by edge-sharing, mainly exists in the melt of KBi(MoO_4_)_2_.

## 1. Introduction

Double molybdates have attracted extensive interest due to their potential applications in various fields of science and technology [1]. Compounds with the general formula ARE(MO_4_)_2_ (A = Li, Na, K, RE = rare earth or Bi, and M = W or Mo) have wide application as hosts for lasers, fluorescence, and scintillating materials [2,3,4,5], due to their excellent chemical durability in air atmosphere, high rare earth ion admittance, large absorption, and emission cross-sections of rare earth ions in their lattice [6,7,8,9,10,11,12,13,14,15,16,17]. Double molybdates of monovalent and trivalent cations have the following three characteristics reported by Isupov [18]: (i) there are many combinations of monovalent (Li, Na, K, Rb, Cs, Tl, Ag) and trivalent (Al, Sc, Fe, Ga, Y, In, La-Lu, Bi) elements in compounds, (ii) the number of crystal types of these compounds is large, and (iii) there are numerous reconstructive phase transitions which differ from the displacement transitions. Regarding the discovery, synthesis, and physicochemical examination of double molybdates with all possible compositions and different cation valences, relevant research began intensively decades ago and has been continuously developing with undiminished interest [19,20,21,22,23].

Much attention has been paid to the rare earth doped bismuth molybdates and tungstates. In particular, double potassium bismuth molybdate KBi(MoO_4_)_2_ are generally used as promising hosts for a variety of luminescent RE^3+^ ions, such as the doped Cr^3+^:KCr_x_Bi_1-x_(MoO_4_)_2_ system [24,25,26]. It was reported that the KBi(MoO_4_)_2_ ceramic showed a distorted scheelite structure and a very low sintering temperature around 903 K [27]. Infrared and Raman spectroscopy were used by Hanuza et al. [28] to obtain the distribution of vibrational levels, the symmetry, and assignment to the respective normal modes of KBi(MoO_4_)_2_ crystal. The transformation of *α*-KBi(MoO_4_)_2_ into the disordered CaWO_4_ structure took place continuously and was accompanied by a very weak endothermic effect [29]. High-temperature melt is the mother liquor of crystal growth; however, the structure of double molybdate melt still remains unknown.

This study attempts to derive the structure information of the melt of KBi(MoO_4_)_2_ from high-temperature Raman spectroscopy, mainly including the structural units in atomic scale and the existing form of multi-molecular clusters structure. In the present paper, we report the temperature-dependent Raman spectroscopy studies of polycrystalline KBi(MoO_4_)_2_ from room temperature to 1073 K in order to obtain information on the structural changes that occur in this material. High-temperature X-ray diffraction (XRD) was also carried out to characterize the thermal stability of the room temperature phase. Quantum chemistry ab initio calculation was carried out to explore the structure of KBi(MoO_4_)_2_ in the molten state.

## 2. Materials and Methods

The starting materials of K_2_CO_3_, Bi_2_O_3,_ and MoO_3_ (analytically pure from Sinopharm Chemical Reagent Co., Ltd., Shanghai, China) were used in the experiment without any purification or treatment. The synthesis of KBi(MoO_4_)_2_ compound was achieved via the melting method, in which the stoichiometric ratio of the original reagents was mixed, uniformly ground, and then heated in a platinum crucible. The temperature control settings are shown in Table 1.

The phase identification was performed using a D8 Advance diffractometer (Bruker AXS, Karlsruhe, Germany) with Cu K*α* radiation in the Bragg–Brentano geometry mode. Temperature-dependent XRD data were collected from room temperature (RT) up to 933 K under air atmosphere in the 2θ range of 5–70° with a step size of 0.016° and an acquisition time of 0.5 s/step. The powder sample was placed in a platinum-lined corundum sample holder and then heated in a high-temperature chamber (HTK 1200N, Anton Paar, Austria). The collected diffraction data were analyzed using the JADE 6.0 software with the JCPDS-ICDD Powder Diffraction File database.

In situ high-temperature Raman spectra of KBi(MoO_4_)_2_ crystal were recorded with a laser confocal Raman spectrometer (LabRAM HR800, Horiba Jobin Y’von, France), which was equipped with an intensity charge coupled device (ICCD) detector for signal enhancement at high temperature. The microscopic heating furnace (Linkam, TS1500, Tadworth, UK) was used to achieve the heating and cooling (the rate controlled at 5 K/min) of the sample in the temperature range from RT to 1073 K with a precision of ±1 K. Each Raman spectrum was collected after keeping the sample for 10 min at the given temperature, which was helpful for elimination of the hysteresis of structural changes induced by temperature. The 532 nm line of a Q-switch pulsed SHG-Nd:YAG laser (Coherent, Santa Clara, CA, USA) was used as an excitation source. In this study, the slits were set for a resolution of about 1 cm^−1^.

In order to explore the melt structure of KBi(MoO_4_)_2_, a series of typical structural units and their multi-molecular clusters structure were designed. Ab initio calculation, a powerful quantum chemistry program for studying finite scale systems [30,31], was performed to study the short-range ordered clusters structure and their properties dependent on this scale of order. The geometry configuration of the designed structural models in the melt of KBi(MoO_4_)_2_ was first optimized before simulating their vibrational Raman spectra using the Gaussian 09 software package. A pseudopotential basis set of LanL2DZ [32,33] and the method of Restricted Hartree–Fock (RHF) [34] were adopted. The single point energy of the cluster structure models with the singlet spin was selected to obtain the ground state.

## 3. Results and Discussion

### 3.1. Temperature-Dependent XRD Spectra

At room temperature and in atmospheric pressure, the crystallization of KBi(MoO_4_)_2_ is monoclinic belonging to the space group *P*2_1_/*c* (*C*_2*h*_^5^), with twelve molecules in the unit cell. It is isostructural with *α*-KSm(MoO_4_)_2_ for which *a* = 16.69, *b* = 23.85, *c* = 5.30, and *β* = 91.3° [28,35]. Some studies have indicated that at temperatures close to the melting or decomposition point, most double molybdates show the tetragonal scheelite structure with a centrosymmetric space group (SG) *I*4_1_/*a* (No. 88) [17]. The structure of KBi(MoO_4_)_2_ is reported to be similar to the distorted scheelite-type (CaWO_4_) structure [36].

**Figure 1:**
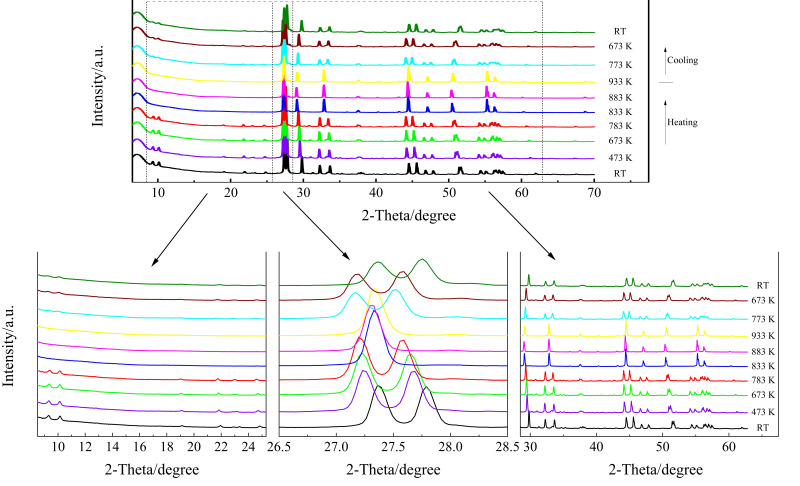
High-temperature XRD patterns of KBi(MoO_4_)_2_, where RT stands for room temperature.

High-temperature XRD of the KBi(MoO_4_)_2_ was performed in order to examine the thermal stability of the monoclinic phase, as is shown in Figure 1. Upon heating, nearly all the diffraction peaks shifted to lower Bragg angles. The XRD pattern changed significantly from 783 K to 833 K, especially the disappearance of the peaks in the 2θ range of 27.2–27.5°, 32–34°, 44–48°, and 54–57°, and the new appearance of the peaks at 27.3°, 32.8°, and 44.5°, which is recognized as the characteristics of the scheelite tetragonal structure. In the low Bragg angle range (<25°), the diffraction peaks disappeared completely at 833 K. That is, as the temperature increased, the split peaks that represent the monoclinic symmetry gradually merged into one, and the ratio of the peak intensities changed with the transformation into a tetragonal structure. The splitting gradually disappeared toward 833 K, and the structure became fully tetragonal with the *I*4_1_/*a* space group. The order–disorder transformation of Bi^3+^ and K^+^ cations gradually occurred in the crystal structure. Some earlier studies indicated that the low-temperature monoclinic phase of KBi(MoO_4_)_2_ continuously transforms to the high-temperature disordered tetragonal scheelite structure in the temperature range ΔT ≥ 150 K and is completed at 933 K, accompanied by a very weak endothermic effect [29,37]. The observed diffraction change from monoclinic to tetragonal is consistent with those reported data. After the temperature decreased to 773 K, a diffraction pattern of monoclinic appeared, but there was still a certain difference from the diffraction spectrum of 783 K. This is probably because the phase structure could not be completely restored due to the faster cooling rate (10 K/min). Therefore, it can be inferred that the phase transition from monoclinic to tetragonal may be reversible at about 773 K when the cooling rate is slow enough. After cooling down to room temperature, the diffraction pattern showed monoclinic reflections. However, the positions of the diffraction peaks after cooling to room temperature had a shift, the intensities of the peaks were lower, and the half-widths of the peaks became larger than those at room temperature before heating. This demonstrates that the monoclinic structure was reobtained, but not completely.

### 3.2. Temperature-Dependent Raman Spectra

For the monoclinic KBi(MoO_4_)_2_, according to the factor group analysis (FGA), the 432 vibrational modes were predicted: *Γ* = 108*A_g_* + 108*B_g_* + 108*A_u_* + 108*B_u_* where *A_u_* + 2*B_u_* are acoustic modes. There are 429 optical zonecenter modes, 216 of which are Raman-active only and 213 of which are IR-active only. It is reported that the predicted vibrational modes were distributed among 54*A_g_* + 54*B_g_* + 54*A_u_* + 54*B_u_* internal modes, 18*A_g_* + 18*B_g_* + 18*A_u_* + 18*B_u_* librational modes, and 36*A_g_* + 36*B_g_* + 35*A_u_* + 34*B_u_* translational modes [28]. According to selection rules, only *A_g_* and *B_g_* are Raman-active.

**Figure 2:**
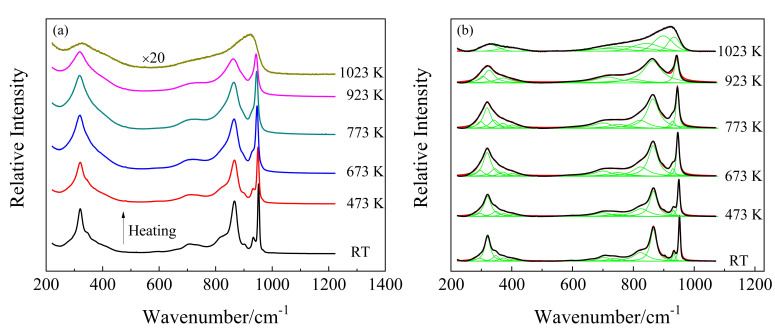
(**a**) Temperature-dependent Raman spectra of polycrystalline KBi(MoO_4_)_2_ from room temperature to 1023 K; (**b**) deconvolution of Raman spectra by Gaussian (1023 K) and Lorentz (from RT to 923 K) function as a function of temperature.

Raman spectra of KBi(MoO_4_)_2_ crystal at room temperature are present in Figure 2. It is obvious that the number of vibrational modes observed is much smaller than that predicted for the KBi(MoO_4_)_2_, which is caused by the polycrystalline samples in the measurement. When it comes to solid-state Raman band distribution, a more appropriate distinction is made between internal and external lattice vibration modes. The most intense lines at 952 and 866 cm^−1^ are attributed to the symmetric and asymmetric stretching vibrations of MoO_4_, respectively. The Raman band in the middle frequency region of 580–750 cm^−1^ is characteristic of the vibrations of double MoO_2_Mo and single bridge MoOMo, which formed due to the intermolecular interactions that exist in the unit cell. The assignment of the major Raman vibrational modes of monoclinic KBi(MoO_4_)_2_ is displayed in Table 2.

**Figure 3:**
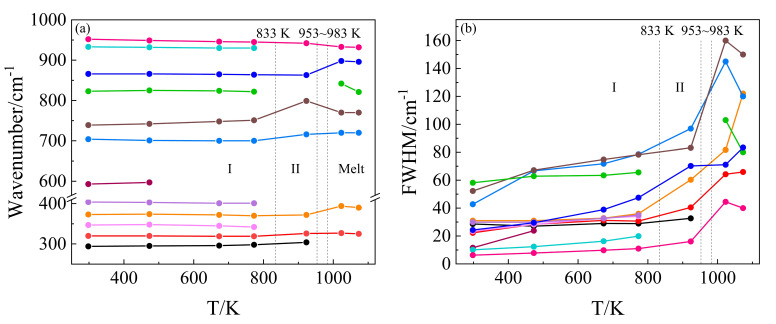
(**a**) Wavenumber versus temperature and (**b**) full width at half maximum (FWHM) versus temperature plot for the major stretching and bending vibrational modes of KBi(MoO_4_)_2_. The vertical dashed lines indicate the reported temperature [27,37] at which the phase transition (phase I: monoclinic, phase II: tetragonal, and the melting point of 953–983 K) takes place.

In situ high-temperature Raman spectra of KBi(MoO_4_)_2_ from room temperature to 1023 K in the wavenumber range of 200–1200 cm^−1^ are shown in Figure 2a. As the temperature increased from room temperature to 923 K, the overall line shapes of the Raman spectra had no obvious change, whereas the full width at half maximum (FWHM) of nearly all the Raman peaks increased and their intensities decreased, which is shown more clearly in Figure 3b. With temperature increasing from RT to 773 K, the adjacent weak and shoulder Raman peaks merged with the major one. It can be seen from Figure 3a that the modes located at 347, 373, 404, 704, 823, 866, 933, and 952 cm^−1^ exhibited a decrease in wavenumber, whereas the modes of 294, 593, and 739 cm^−1^ showed a linear increase with increasing temperature. The structure gradually relaxed with broadening distribution of atomic bond distances and bond angles due to thermal expansion and thermal disorder. With a further temperature increase to 923 K, the vibrational modes of 342, 401, 822, and 930 cm^−1^ (monoclinic phase at 773 K) disappeared, while the modes of 298, 319, 700, and 751 cm^−1^ increased obviously in wavenumber. The Raman response was still very well-defined at 923 K, but it was suddenly strongly diffused at 1023 K. At first sight, such spectral evolution should be related to structural phase transitions. Combined with the high-temperature XRD results, it can be determined that at 833 K the monoclinic phase of KBi(MoO_4_)_2_ to the tetragonal transformation occurred. A similar pseudo-tetragonal lattice distortion appearing in KBi(MoO_4_)_2_, which was attributed to the ordering of the K^+^ and Bi^3+^ ions [37], may explain the slow and continuous change of Raman spectra before this transition. Figure 2b demonstrates the deconvolution of Raman spectra corresponding to the experimental temperatures by Gaussian and Lorentz function for the molten spectrum and the other spectra, respectively. The spectrum of the KBi(MoO_4_)_2_ melt was deconvoluted into seven Gaussian Raman peaks at 1023 K. The dramatic changes in the Raman spectra from 923 K to 1023 K were due to the solid–liquid phase transformation, and the tetragonal crystal structure was destroyed and in a completely molten state.

### 3.3. Structure of Molten KBi(MoO_4_)_2_

**Figure 4:**
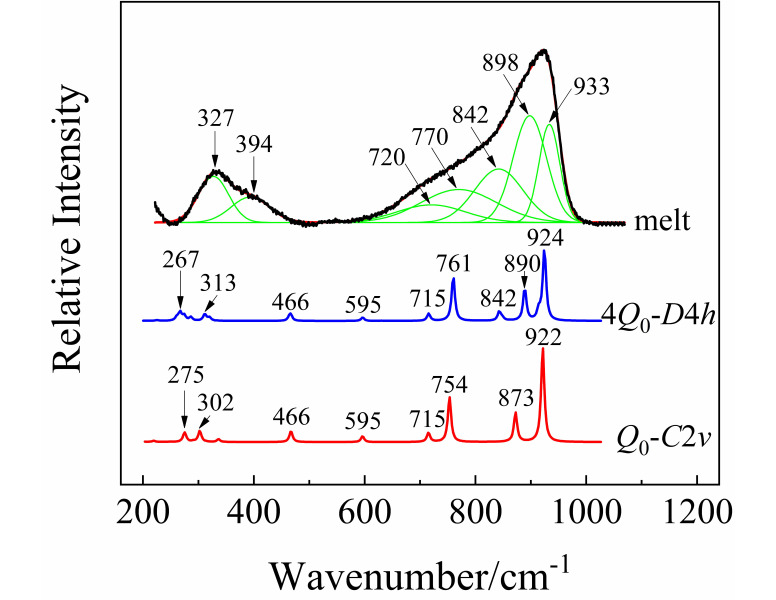
The deconvolution of Raman spectrum for the melt of KBi(MoO_4_)_2_ at 1023 K by Gaussian function, and the calculated Raman spectra by quantum chemistry ab initio calculation, where *Q*_0_ and 4*Q*_0_ represent the structural unit and the corresponding multi-molecular clusters structure containing four structural units.

The Raman spectrum of the molten KBi(MoO_4_)_2_ at 1023 K is shown in Figure 4. The whole envelope was deconvoluted by Gaussian function after subtracting the baseline of the spectrum. The anion motifs of isolated [MoO_4_]^2−^ tetrahedra have been reported to exist in the melt of alkali metal monomolybdates [38,39]. KBi(MoO_4_)_2_ has the same chemical ratio as K_2_MoO_4_; however, their band shapes and positions are quite different. This indicates that the melt structure of KBi(MoO_4_)_2_ cannot be the simple monomer of [MoO_4_]^2−^. Nevertheless, on the basis of the distribution of different structural units previously studied [40], the six coordinated [MoO_6_]^6−^ cannot be the primary structure in the melt of KBi(MoO_4_)_2_. From the positions of the deconvoluted Raman peaks, the four coordinated tetrahedral [MoO_4_]^2−^ can be confirmed to be present in the melt.

**Figure 5:**
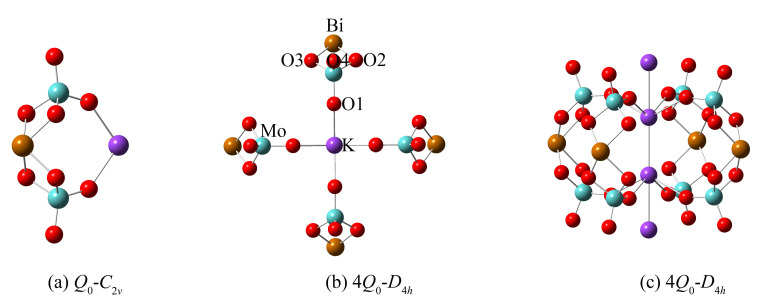
(**a**) The designed structural units and (**b**,**c**) the corresponding multi-molecular clusters structure for the ab initio calculation of molten Raman spectra of KBi(MoO_4_)_2_. Here, (**b**,**c**) represent the same cluster structure containing four molecules viewed from the in-plane and out-of-plane directions. The elements are labeled (Mo, light blue; K, violet; Bi, light brown; O, red) in the diagram of (**b**).

In order to further examine the structural unit and its multi-molecular clusters structure in the melt of KBi(MoO_4_)_2_, quantum chemistry ab initio calculation was performed for the optimization and simulation of the vibrational properties of the designed structure. Considering the large electronegativity of the heavier Bi atom, the [MoO_4_]^2−^ group is more likely to be coordinated to Bi^3+^ than K^+^. Therefore, a model of “double-center” structure with K^+^ acting as the charge compensation was proposed and illustrated in Figure 5a. Due to the strong attraction of Bi to electrons, it will compete for the non-bridging oxygens around Mo atoms, thereby increasing the number of bridging oxygens in the system. Note that Bi is a sub-center, while Mo is still the center of the entire structural system. The oxygen atoms coordinated to Bi are also shared by the [MoO_4_]^2−^ tetrahedra. Taking into account the effect of multi-molecular cluster structure, the corresponding multi-molecular cluster containing four structural units was also built and is exhibited in Figure 5b,c. The calculated Raman spectra of the cluster structure models by ab initio calculation are shown in Figure 4 after being corrected by a wavenumber scaling factor of 0.8555 and normalizing the intensity. Through comparative analysis, the calculated Raman frequencies and intensities are consistent with the data measured experimentally.

As can be seen from Figure 5, the structure models of anion motifs (a) represent a monomer (*Q*_0_) molecule of KBi(MoO_4_)_2_ with *C*_2*v*_ symmetry, and (b,c) represent the multi-molecular cluster structure with higher *D*_4*h*_ symmetry which consists of four monomers (4*Q*_0_). In the anionic multi-molecular cluster structure, the environment of each *Q*_0_ is equivalent. Figure 4 shows the calculated Raman spectra of a different number of model clusters of KBi(MoO_4_)_2_. The calculation result demonstrates that as the number of model clusters increases, the frequency of the characteristic peaks shifts toward a high wavenumber, which has been observed earlier caused by the multi-molecular cluster effect. Therefore, considering the environmental effects of cations, the more reliable multi-molecular cluster structure is closer to the real structure of the melt of KBi(MoO_4_)_2_. Through the combination of the experimental and calculation data, it is proved that the structure of the [Bi(MoO_4_)_2_]^−^ monomer with Mo^6+^ centers and Bi^3+^ sub-centers primarily presents in the melt of KBi(MoO_4_)_2_ molybdate and is distributed in a short-range ordered and long-range disordered state.

In Figure 4, the fitted Raman peak at 933 cm^−1^ originates from the symmetric stretching vibrations of non-bridging oxygens of Mo-O_4_. The peaks located at 898 and 842 cm^−1^ are attributed to the asymmetric stretching vibrations of non-bridging oxygens of Mo-O_1_. The peak position at 761 cm^−1^ from calculation clearly shows the vibrations of the Bi-O bond with an oxygen coordination number of 4. The wavenumber region of 450–770 cm^−1^ mainly involves the vibrations of Bi-O in the structure. Table 3 lists the experimental and calculated wavenumbers of the major vibrational modes and their assignment in the melt of KBi(MoO_4_)_2_. For instance, the peak at 770 cm^−1^ was assigned to the symmetric scissor vibrations of Bi-O_2_ and Bi-O_3_, whereas the asymmetric scissor vibrations of Bi-O are around 720 cm^−1^. The peaks at about 394 and 327 cm^−1^ are caused by the asymmetric stretching and wagging vibrations of Mo-O in the clusters structure.

## 4. Conclusions

In situ high-temperature Raman spectroscopy and quantum chemistry ab initio calculation were applied to investigate the structure present in the melt of KBi(MoO_4_)_2_ molybdates. The temperature-induced phase transition from monoclinic to the scheelite tetragonal structure was observed to occur at 833 K by temperature-dependent XRD. The calculated results via the ab initio method are in good agreement with the experimental Raman data, demonstrating the reliability of the assignment of the observed Raman bands for the molten KBi(MoO_4_)_2_ at 1023 K. The isolated structure of [Bi(MoO_4_)_2_]^−^ monomer anion, in which the oxygen coordination numbers of Mo and Bi atoms are both four and connected to each other by edge-sharing, is confirmed to exist primarily in the melt of KBi(MoO_4_)_2_. The study on the melt structure of KBi(MoO_4_)_2_ makes it possible to examine clusters structure composed of different complexes and M^z+^ cations and opens up a new way for the exploration of the development of novel laser host materials.

## Figures and Tables

**Table 1:** The temperature control settings used for the synthesis of KBi(MoO_4_)_2_ crystal.

Temperature Point/K	Holding Time/min	Heating or Cooling Rate/K·min^−1^
298	-	4.92
593	60	1.14
723	30	
823	30	
923	60	
1023	60	
1073	60	
473	-	The melt cooled down slowly (0.25 K/min) to 473 K, and then cooled in the furnace to room temperature.

**Table 2:** The assignment of major vibrational modes of crystalline KBi(MoO_4_)_2_.

Wavenumber/cm^−1^		Type of Vibration
*ν*_exp._	*ν*_ref._ [23]	
952	948	symmetric stretching vibrations of MoO_4_
933	929	
902	898	
866	863	asymmetric stretching vibrations of MoO_4_ with stretching vibrations of Mo_2_O_8_ bridge
823	815, 830	
739	736	
704	704	
658	-	
593	580	
404	-	asymmetric bending vibrations of MoO_4_
373	376, 365	
347	343	
320	320	symmetric bending vibrations of MoO_4_
294	282	

**Table 3:** The attribution of major vibrational modes in the melt of KBi(MoO_4_)_2_.

Wavenumber/cm^−1^		Type of Vibration
*ν*_exp._	*ν*_cal._	
933	924	symmetric stretching vibrations of Mo-O_4_
898	890	asymmetric stretching vibrations of Mo-O_1_
842	842	
770	761	symmetric scissor vibrations of Bi-O_2_ and Bi-O_3_
720	715	asymmetric scissor vibrations of Bi-O
-	595	rocking vibrations of Bi-O
-	466	symmetric stretching vibrations of Bi-O_2_ and Bi-O_3_
394	313	asymmetric stretching and wagging vibrations of Mo-O
327	267	
